# Diabetes mitocondrial: de la sospecha en atención primaria al abordaje multidisciplinar familiar

**DOI:** 10.1016/j.aprim.2025.103370

**Published:** 2025-10-06

**Authors:** Blanca Adriana Macías Martínez, Alba Dalmau Vila, Marta Hernández García, Maria Antonia Lafarga Giribets

**Affiliations:** a ABS Bordeta-Magraners, Institut Català de la Salut, Lleida, España; b Servei d’Endocrinologia i Nutrició, Hospital Universitari Arnau de Vilanova, Lleida, España; c Institut de Recerca Biomèdica Lleida (IRB Lleida), Universitat de Lleida, Lleida, España; d Departament de Medicina i Cirurgia, Facultat de Medicina, Universitat de Lleida, Lleida, España

La mutación del DNA mitocondrial (mtDNA) es una de las causas más frecuentes de enfermedades hereditarias^1^, con tres particularidades: a) herencia principalmente por vía materna; b) la mutación patogénica puede afectar en diversa proporción a los diferentes tejidos, lo que se conoce como heteroplasmia, y dificulta el diagnóstico, y c) las copias de mtDNA varían según el tejido y a lo largo del tiempo^1^.

La prevalencia de enfermedades mitocondriales varia de 1/5.000 niños hasta 1/8.000 adultos^2^. La mutación 3243A>G en el gen *MT-TL1* es la más frecuente^3^. Principalmente se manifiesta en órganos con alto consumo de energía, como el sistema nervioso, corazón y páncreas, aunque puede afectar prácticamente a cualquier tejido, sistema y órgano^4^. El fenotipo más común (30%) es el síndrome MIDD (diabetes mellitus [DM] de herencia materna con hipoacusia neurosensorial) y un 10% se puede presentar como MELAS (miopatía mitocondrial, encefalopatía, acidosis láctica y episodios que simulan ictus), acompañado o no de DM^3^. Se ha acuñado el término «portador latente» para aquellos pacientes con mutación mitocondrial pero sin síntomas clínicos^3^.

El MIDD es el fenotipo más común del sistema endocrino, y el 85% de los pacientes presentan la mutación A3243^4^. La DM suele iniciarse de manera similar a la DM2 pero en edades más jóvenes (30-40 años de media). Puede ser tratada inicialmente con dieta y sulfonilureas. La metformina está contraindicada por riesgo de acidosis láctica. Generalmente presentan un declive rápido de la función de células β y necesitan insulina en 2-4 años, dependiendo de los niveles de heteroplasmia. Los pacientes suelen presentar un índice de masa corporal (IMC) normal/bajo^4^, ^5^.

En las enfermedades mitocondriales el diagnóstico requiere un alto grado de sospecha. El hecho de que un paciente presente DM2 con IMC normal e hipoacusia es un factor clave para derivarlo a un servicio especializado^3^. El diagnóstico se establece con la secuenciación de mtDNA en sangre u orina, aunque en ocasiones es necesario realizar una biopsia de músculo esquelético^6^ con biopsia de piel. Una vez localizado el caso índice, se debe extender el estudio a familiares por rama materna.

No existe una terapia curativa por el momento, aunque actualmente se realizan diversos ensayos: combinado de vitaminas, antioxidantes y cofactores, modificaciones adicionales basadas en la etiología genética, fenotipos y hallazgos bioquímicos. A las mujeres portadoras de la mutación mitocondrial se les ofrece consejo genético y opciones reproductivas^6^.

Favorecido por la consanguinidad, existen familias estudiadas en Países Bajos, Japón, Australia y Turquía^4^, ^5^ afectas por dichos síndromes.

En nuestra región recogimos los casos de dos familias con alta penetración de síndrome MELAS y MIDD, en los que el diagnostico se realizó por la alta sospecha del caso índice con diabetes y sordera. El probando de la primera familia (fig. 1a) presentaba DM desde los 23 años, insulinotratada y con mal control, además de episodios *ictus-like*, DM gestacional previa y fibrilación auricular. El caso índice de la familia 2 (fig. 1b) presentaba DM insulinotratada desde los 32 años, DM gestacional previa y síntomas digestivos, neumológicos, cardiológicos y tiroideos. Estudiados los familiares, destaca la alta penetrancia de la mutación. Los pacientes familiares presentaban, además de DM, taquicardias/arritmias, déficit intelectual, pérdida de agudeza visual, mialgias, hipotiroidismo, anemia ferropénica y retrasos madurativos en el caso de los niños.

**Figura 1:**
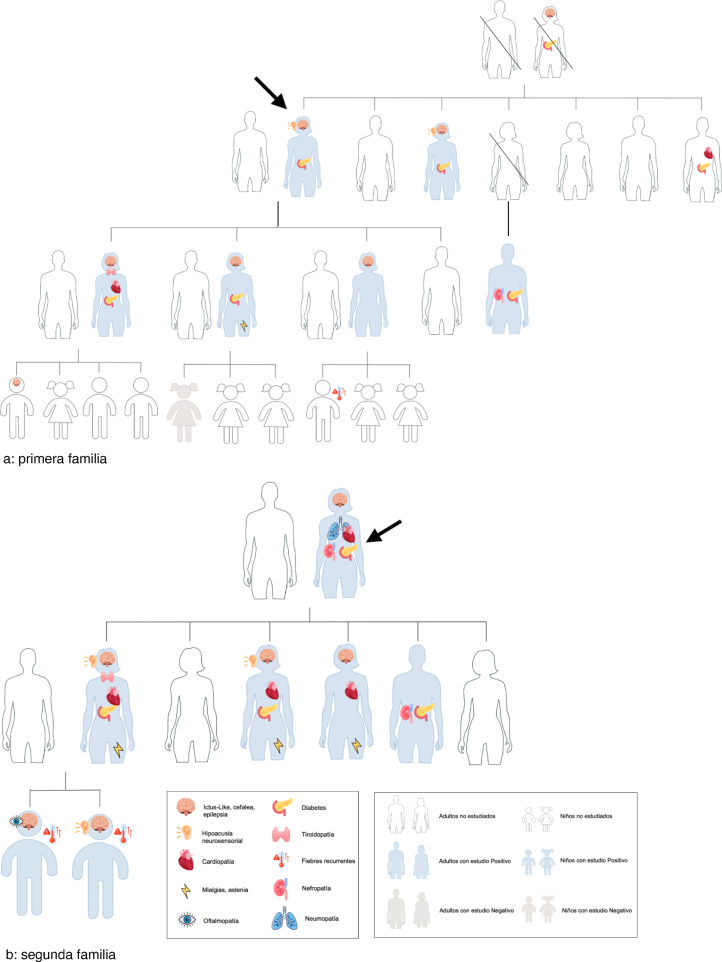
Genograma de los pacientes estudiados. a) primera familia; b) segunda familia.

Se necesita, pues, un alto índice de sospecha por parte del médico de familia para diagnosticar esta entidad. La sospecharemos en familias con pacientes diagnosticados de DM2 relativamente jóvenes (20-60 años), con herencia por vía materna, sin síndrome metabólico e IMC dentro de la normalidad, con otras alteraciones asociadas como hipoacusia, miopatía, neuropatía óptica y/o crisis comiciales. El hecho de padecer una DM gestacional en pacientes sin obesidad/síndrome metabólico también es relevante en nuestras familias.

## Financiación

Este estudio no recibió ningún tipo de financiación.

## Conflicto de intereses

Los autores declaran no tener ningún conflicto de intereses.

## Consideraciones éticas

Los autores declaran que se han seguido los protocolos establecidos por sus respectivos centros sanitarios para acceder a los datos de las historias clínicas a los fines de poder realizar este tipo de publicación con finalidad de investigación/divulgación para la comunidad científica.
